# Breastfeeding Supports and Services in Rural Hawaii: Perspectives of Community Healthcare Workers

**DOI:** 10.1155/2017/6041462

**Published:** 2017-01-11

**Authors:** Jeanie L. Flood

**Affiliations:** University of Hawai‘i at Hilo, 200 W Kawili Street, UCB 237, Hilo, HI 96720, USA

## Abstract

*Background*. In the state of Hawaii, breastfeeding initiation rates are higher than the national average but fall below target rates for duration. Accessing breastfeeding support services is challenging for mothers living in rural areas of the state. Healthcare workers (HCWs) working with mothers and infants are in a key position to encourage and support breastfeeding efforts. The purpose of this study is to gain a better understanding of a Hawaiian community's (specifically Hilo, Hawai‘i) breastfeeding service and support issues.* Method*. The qualitative study design utilized was a focused ethnography. This approach was used to gather data from participant HCWs (*N* = 23) about their individual or shared experience(s) about the breastfeeding supports and services available in their community. An iterative process of coding and categorizing the data followed by conceptual abstraction into patterns was completed.* Results*. Three patterns emerged from the qualitative interviews:* Operating within Constraints of the Particular Environment*,* Coexisting Messages*, and* Process Interrupted.* Participants identified a number of gaps in breastfeeding services available to their clients including the lack of available lactation consultants and the inconsistent communication between hospital and community providers. A number of implications for practice and further research were suggested within the results and are discussed.

## 1. Introduction

Despite recommendations from government and health organizations, the duration of breastfeeding in the United States (US) is still below desired levels. The* Healthy People 2020* target, increased from the* Healthy People 2010*, is for 81.9% of mothers to initiate breastfeeding, 60.5% to breastfeed for at least six months postpartum, and 34.1% to continue breastfeeding through 12 months after delivery [1]. According to the USDHHS* Blueprint for Action on Breastfeeding*, one of the major goals of the* Healthy People *targets was to “eliminate health disparities among different segments of the population” ([2], p. 8). Despite substantial increases in breastfeeding rates in some areas of the US, racial and ethnic disparities are widespread [2]. In 2006 only 59.7% of Asian and Pacific Islander (PI) infants were ever breastfed with the rate dropping to 29.7% at six months. It is an important public health goal to increase breastfeeding rates “particularly among racial and ethnic groups who are less likely to initiate and sustain breastfeeding for the first year” ([2], p. 9).

State of Hawai‘i data from the Pregnancy Risk Assessment Monitoring System (PRAMS) indicate that while the statewide rate of mothers initiating breastfeeding has steadily increased from 89.1% in 2000 to 92% in 2006, only 36.4% of women who initiated breastfeeding exclusively breastfed for eight weeks, and 20.7% of women breastfed less than eight weeks [3]. Some of the characteristics of women who were less likely to breastfeed included being Native Hawaiian (NH), younger (i.e., less than 20 years of age), and less educated (i.e., did not graduate from high school) and living in Hawai‘i County (i.e., the island of Hawai‘i) [3].

An important factor that may be influencing breastfeeding rates reported for the island of Hawai‘i is access to breastfeeding support. The availability of information and resources that support breastfeeding infants may help women to initiate and to prolong duration of breastfeeding [4] which in turn will enable vulnerable populations to receive the health benefits that are associated with breastfeeding.

The AAP emphasizes the essential role of pediatricians and healthcare providers (HCPs) in promoting, protecting, and supporting breastfeeding and makes specific recommendations about breastfeeding policies. According to Pillitteri [5], support from healthcare personnel is important to help a woman feel secure enough to be able to relax and breastfeed.

In a news release, the* Surgeon General* of the US released a* Call to Action* to support breastfeeding [6]. According to the news release, the following was stated.

Many mothers who attempt to breastfeed say several factors impede their efforts, such as a lack of support at home; absence of family members who have experience with breastfeeding; a lack of breastfeeding information from healthcare clinicians; a lack of time and privacy to breastfeed or express milk at the workplace; and an inability to connect with other breastfeeding mothers in their communities.

The* Call to Action* identifies specific ways that communities, families, and health professionals can increase support for breastfeeding.

## 2. Background

An analysis of recent data from the CDC focusing on breastfeeding support revealed that the state of Hawai‘i scored lowest in the nation for breastfeeding support after hospital discharge [7]. Moreover, the availability of breastfeeding services may be more limited in rural settings in the state. The geographic location of the island of Hawai‘i, which is classified as a rural region in the state of the same name, has particular difficulties with access to healthcare which can result in suboptimal services. Currently, breastfeeding supports and service needs for the island of Hawai‘i are unknown. This complicates the ability of pregnant or new mothers living on this island to access quality healthcare services, including breastfeeding services. The perspectives of the healthcare workers (HCWs) working with breastfeeding women can provide an opportunity to understand why women choose or  do not choose to breastfeed and are an important element to include in any intervention to support breastfeeding. HCWs in this island community include a wide range of practitioners including physicians, nurses, medical assistants, lactation consultants, and doulas. The purpose of this study is to gain a better understanding of a Hawaiian community's (specifically Hilo, Hawai‘i) breastfeeding service and support issues.

## 3. Conceptual Framework

The social ecological model with an emic perspective is the conceptual framework for this exploration. The emic perspective seeks to understand the insider's perceptions about a phenomenon. Research using this approach is driven by the views of the participants [8].

The social ecological model evolved through the work of a number of researchers.

One researcher whose work has contributed to the social ecological model is Urie Bronfenbrenner. Bronfenbrenner was a psychologist concerned with issues of child development and is credited with the development of the Ecological Systems Theory. Bronfenbrenner [9] described the “ecology of the family” as the context for human development. His research focused on the relationship between individuals and their environment and included the idea of contextual relevance. Bronfenbrenner [10] saw the influences on child behavior as a series of nested layers where each layer had an impact on the subsequent level. For example, family is embedded within and influenced by the physical environment.

Daniel Stokols, a researcher at University of California, Irvine School of Social Ecology, created the Social Ecology Model, which is similar to the Ecological Systems Theory. This model is represented by a series of overlapping circles with each circle representing a layer or component of the model and each having an impact on the individual [11]. According to Stokols [12], all levels of social ecological analyses are characterized by a broad contextual scope with a focus on the interaction between people and their environments that may explain health issues or provide the basis for interventions that may enhance personal and community wellbeing. The social ecological model although common in healthcare research must be modified to each research focus and population group [13].

**Figure 1:**
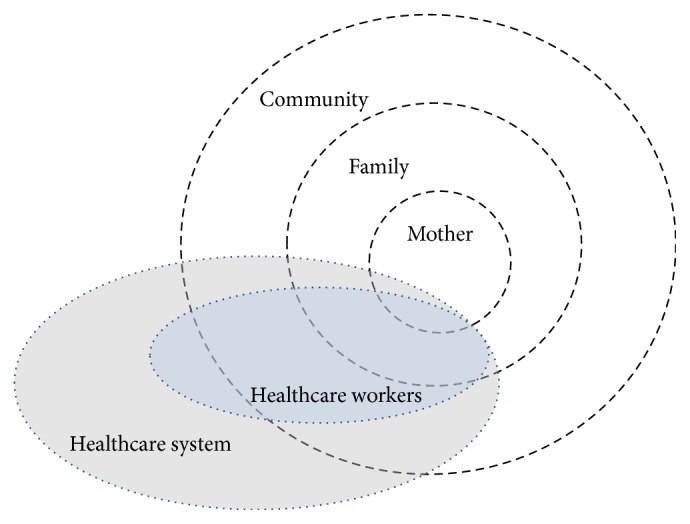
Conceptual diagram of the different layers of influence on breastfeeding patterns. Note: the size of the circle does not correlate with the potential influence that each category may exert on breastfeeding patterns.

For the purpose of this study, the social ecological model was tailored to focus on the different layers of influence on breastfeeding patterns including the mother, the family, the community, HCWs working with the community, and the larger healthcare system (see Figure 1).

Factors that influence decisions about infant feeding include those of the mother [14], the family [15, 16], and the community and larger society [17]. HCWs, a part of the healthcare system, influence community, family, and mothers and may contribute to choices mothers make regarding breastfeeding [18]. This approach offers the opportunity for increased understanding of the sociocultural, psychological and environmental influences on breastfeeding supports and services available to families in this context [12].

### 3.1. Aim

The goal of this study was to explore HCWs perceptions about existing breastfeeding supports and services in a federally designated rural community on the island of Hawai‘i, specifically Hilo. The specific aim that the study addressed is to describe HCWs' understanding of community breastfeeding supports and services that exist within Hilo, Hawai‘i.

## 4. Methodology

The research reported in this paper is part of a larger ethnographic study that explored breastfeeding supports and services in Hilo on the island of Hawai‘i. A focused ethnography was used in order to gather data from participants about their individual or shared experience(s) with breastfeeding supports and services [19]. This method was chosen to target a specific group of healthcare workers.

### 4.1. Setting

Although a part of the US, the state of Hawaii is approximately 2,500 miles from the US mainland and is located in the center of the Pacific Ocean. The island of Hawai‘i is the largest island geographically in the state with a land mass of 4,028 square miles. It has the second largest county population with 172,547 residents; however, it has the lowest population density per square mile in the state [20]. Hilo is the setting for this study and the largest city on the island of Hawai‘i with a population of 42,916 and is located on the eastern and windward side of the island. The town of Hilo, located in East Hawai‘i, is built surrounding a large deep-water bay, and is the seat of county government. In 1946 the city was nearly obliterated by a tsunami and suffered the effects of another tsunami in 1960 [21]. Hilo is less frequented by tourists than other parts of the island of Hawai‘i, in part, because it is somewhat geographically isolated, surrounded by two active volcanoes, and because it is one of the wettest areas in the US, with an average yearly rainfall of 132 inches. The climate and relative isolation has allowed Hilo to retain a strong sense of local culture. The unique geographic and social-cultural features of Hilo, which include a relatively isolated multicultural island community, contribute to its being an appropriate setting for this exploration. No research could be done in this area without considering the ethnic diversity of the community. As a result of the indigenous population who first settled this land and the influx of immigrants who initially came to work on sugar plantations, it is no surprise that Hilo is home to a diverse population which continues today. Access to perinatal services on Hawai‘i Island is complicated by limited providers, the rural landscape, and socioeconomic issues among other factors. Consequently, breastfeeding and support services, components of perinatal services, may be inaccessible or unavailable on the island.

### 4.2. Sample

HCWs who work with childbearing women in Hilo were the target population for enrollment in this study. The researcher systematically identified the key stakeholders in the community through established relationships and recommendations of known health professionals. An effort was made to seek participants working with mothers and infants in a variety of settings in the community. There were no inclusion or exclusion criteria. No attempt was made to determine a participants' views of breastfeeding prior to the study. A combination of purposive and snowball sampling was used to identify potential study participants. These sampling techniques were appropriate for the collection of descriptive data within a specific population where the aim is to describe a particular experience or phenomenon [22]. Sampling continued until saturation was reached (*N* = 23).

### 4.3. Data Collection Procedures

Approval of the study protocol was obtained from the Institutional Review Board (IRB) at the University of Hawai‘i at Mānoa prior to participant recruitment. All potential participants were informed of the purpose, aims, and goals of this research project. If a person agreed to participate, informed consent was obtained prior to their participation in the study. Participants were also informed that they could withdraw from the study at any time. To protect participant anonymity, all personal identifying information (e.g., name) was removed. Demographic information was aggregated and this information did not remain as a part of the participant's interview data.

Data were collected in 2009 and 2010 using tape recorded interviews. Each interview included general questions and prompting to encourage participants to share their understanding of breastfeeding supports and services in their community. Questions encouraged participants to explain or describe their perspectives.

### 4.4. Data Analysis

Following the interview, the audio-taped recordings were sent to a transcriptionist. Following the transcription, each interview was compared with the audio-taped recording and checked for accuracy and completeness and any corrections were made. The interview data were then entered into Atlas.ti (version 5) [23] to facilitate coding and retrieval of data.

To describe HCWs' understanding of community breastfeeding support and services, ethnographic methods were used. This process involved four steps: (1) coding of the content of the interviews; (2) categorizing the data; and (3) identification of patterns. Coding was data driven and inductive with terminology taken from the raw data. Throughout the coding process, a coding decision log was maintained to document any modifications to the code list and a rationale for any changes. Following completion of coding, a code book of the codes and quotations was created to clearly define the code and code definition based on the data.

The second phase of analysis was the identification of categories. During this process, the ecological conceptual framework for the study was ascertained to be an appropriate framework to identify higher themes. The rationale for this approach was the multilayered aspect of how HCWs view their community. The codes and categories  do not exist in isolation but overlap within one another and across the spheres of influence. Using the ecological framework, codes were grouped and regrouped within the spheres of the framework. Miles and Huberman [24] suggest that a structured research design utilizing a tight research framework can increase clarity and focus and help researchers avoid data overload. A methods expert reviewed the analysis.

The final phase of the analysis was the abstraction into higher-level constructs and a taxonomy of categories in the form of a concept map. This form of pattern coding is a way to group the categories into a smaller number of overarching constructs and illustrate relationships among them [24].

### 4.5. Methodological Rigor

Quality in the research process was maintained by the use of the trustworthiness criteria proposed by Lincoln and Guba [25]. This included the creation of an audit trail to document all decisions of the coding and analysis. The researcher maintained a reflexive journal to identify and address any instances of bias. Cross checks by another researcher who is a content and methods expert were completed on at least 25% of the analysis to establish credibility. Transferability was ensured through the establishment of a comprehensive description of the design methodology and triangulation among multiple data sources. Field notes were kept as part of the audit trail.

While it is not necessary for a researcher to be a member of a group under study for research to be culturally sensitive, knowledge of the community is essential [26]. The principal investigator (PI) for this study has lived on the island of Hawai‘i for approximately eleven years. She has resided on both the east and west sides of the island working in the field of education and maternal child health. She has knowledge of the community and many of the customs and cultures of the island. The PI has been an international board certified lactation consultant (IBCLC) for 14 years and has an established interest in breastfeeding support.

## 5. Results

### 5.1. Characteristics of the Sample

The sample (*N* = 23) of HCWs serving mothers and infants in East Hawai‘i ranged in age from 29 to 61 years with a mean of 41.5 years (SD = 9.8). The 23 workers included nurses, dieticians, medical, or nursing assistants. Participants reported living in Hilo from 3 to 48 years (*M* = 21.5). Participants had been working with mothers and infants for an average of 12 years. One participant was a male and the majority of participants were parents who reported a history of breastfeeding their own infants with an average breastfeeding time of 18.4 months.

### 5.2. Patterns

Three patterns emerged from 14 categories and 55 codes. The patterns were* Operating within Constraints of the Particular Environment, Coexisting Messages*, and* Process Interrupted* (see Table 1).

Each pattern is presented as a discrete entity; however, it is assumed that they do overlap, as they represent the whole perspective of the participants. The patterns are defined and described with quotes included to illustrate the voices of the participants.

### 5.3. Operating within Constraints of the Particular Environment

The pattern of* Operating within Constraints of the Particular Environment* (*n* = 584: 44%) is characterized by the practice environment of participants and their clients. HCWs practice/operate within the constraints and limits of their physical and socioeconomic context, including the local healthcare system and geographic island locale. Their clients are also situated within this context.

According to many participants, the place where a mother is situated by experience, demographics, socioeconomics, and lifestyle influence their breastfeeding decisions. Mothers are described as challenged by difficult socioeconomic situations and, despite support from HCWs, find breastfeeding a challenge. From one participant,

*I don't know if you notice but I'm sure a lot of the men are not working, a lot of the women are working and so a lot of the times the woman doesn't want, they just say it's so hard, I have to go to work, try to pump and the father is at home taking care of the child and it's just not as easy.* (Participant #16)

Participants described the age of their clients influencing breastfeeding choices. Most describe breastfeeding as more challenging to younger mothers. From one medical assistant,

*I think maybe the younger mothers, [we] have so many young mothers, I think it's probably part being embarrassed of nursing. You know you're still young yourself and you're exposing your breast. When you're around a young crowd you don't want to just go on the side and nurse. *(Participant #11)

Several participants described young mothers as finding their lifestyle incompatible with breastfeeding. In addition to the challenge of returning to work or school, young mothers' priorities may conflict with breastfeeding. According to one hospital staff nurse,

*like I've had some young moms and I see them come back…they'll tell me, oh, I stopped because they want to go out and party. So I guess a lifestyle a little bit, is depending on the age of the person if they want that level of commitment, you know.* (Participant #6)

Issues of island living and a rural location influence what services and supports are available to the community. Some participants describe how medical services vary within the state and how limited access to healthcare is for rural Hawai‘i County residents. From one community nurse, “When you look in the telephone book at the doctors and the telephone numbers, most of them are in like two or three zip codes and are on Oahu” (Participant #1). Services are also described as varying within the island. According to one WIC worker, “And the fact of being on this island I feel West Hawai‘i is a lot more advanced than the East is. Maybe there is a lot more mainland influence on it? I don't know what it is.” (Participant #18)

In addition to the geographical aspects of island living, the area healthcare structure itself creates hurdles for HCWs in their breastfeeding support efforts. Participants describe issues such as a lack of leadership for breastfeeding promotion and the pervasiveness of formula in the community as challenges to support breastfeeding. From one of the WIC staff,

*everyone confuses each other and maybe this is going to be a great idea to do and it fizzles out, nothing is done. I think we just kind of get it together and be on the same page. That would be so nice. Not only for breastfeeding but like in general, everything I think we're really behind.* (Participant #18)

One of the hospital nurses shared her perspective on the lack of resources at the hospital, “I think it has a lot to do with finances and then also it, also it has to do, I think also has to do with admin” (Participant #9).

Community norms and beliefs about infant feeding are an influence, in particular the use of formula. Many describe the availability of formula as interfering with their breastfeeding support efforts. One program administrator described the role of formula, “One of the deterrents of formula is the cost. If you get it all free than it takes out a major deterrent” (Participant #4). Many describe mixed feedings of breast and formula feeding as a common practice. One nutritional assistant shared how mixed feedings is often a precursor to a discontinuation of breastfeeding.

*They try to do both but the problem is once they start doing both it's so much easier to do the formula that they just stop. I think one of the best things coming, personally, tell a lot of people breastfeed is not give them formula.* (Participant #16)

Another structural hurdle described by participants is the lack of knowledge of the benefits of breastfeeding by the community, described by one worker as a “lack of public awareness.” HCWs identify the need for more community education but express a frustration with no clear pathway to disseminate information. From one hospital staff nurse, “So it starts to make me wonder how much of like the community doesn't get enough education in whole about the benefits of breast feeding to have it be accepted.” (Participant #5). One community provider shared her hope,

*I'd really like to see this community take it on more, however that may go. I really, education is key empowering women to believe in it and so they advocate for themselves. I think that's what will really change the environment.* (Participant #4)

HCWs describe how they support breastfeeding in their own practice within the constraints of their environment. They also share a frustration with some of the routines in other settings and describe how it impedes their breastfeeding support efforts. Hospital practices and the hospital experience were identified by many participants, including some who work in the hospital, as unsupportive to breastfeeding. As one outreach worker shared,

*I can give them all the best information about breast feeding… but if they're still sitting on that fence and they go into give birth and maybe they had a very long and hard labor and a nurse comes in and isn't very, you know, like trying to…[the mother says] just give me the bottle. And they kind of give the bottle, you know when they bring in that cradle the bottles are underneath….* (Participant #3)

Some participants report hearing negative things from their clients about how they were unsupported at the hospital but express some skepticism about the accuracy of their client's perceptions. From one nutritional assistant, “Being here listening to all the girls that come in I don't know what the exact truth is because I'm only taking their word but a lot will say at the hospital they gave my baby a bottle.” (Participant #16). One staff nurse described how the expectations of parents are a contributing factor,

*here it's more of the expectation is you're going to take my baby for the night, right? So we're trying to make the change already at the hospital to the babies stay in with the mom's. The community kind of like what is going on here? I wanted to rest.* (Participant #5)

Early discharge from hospital to home, described as being a common practice, prevents mothers from getting support and information about breastfeeding. From one pediatrician, “Then I think at our hospital level our culture here [is] to discharge mom's pretty early” (Participant #8). Following discharge, many participants described not knowing what sort of follow-up mothers were receiving. Those working with mothers in the prenatal period were not always aware of when mothers deliver and may not see them until several weeks after discharge. From one support group leader,

*and the crazy thing is that I don't even know what kind of follow-up they're receiving at the pediatrician's office versus so now we're inundated in our pediatricians who are already over loaded with all these, you know, breast feeding issues and I don't really know how they're handling them.* (Participant #5)

In addition to identifying the challenges to support breastfeeding, participants describe how they make the most of what exists. The use of and referral to breast pumps and a local support group were described by most participants as part of their practice as well as the distribution of handouts on breastfeeding that are available at their worksites. More inconsistent was the ability to refer for more intensive lactation services or have a reliable phone resource for mothers with questions. Only one work setting had the services of an IBCLC available for their clients. Several described how they could refer mothers to an IBCLC outside of the community but that many of their clients are too constrained by time and money to utilize that option. From one community nurse,

*I've had three ladies now that had enough money to hire the lactation consultant [out of the community, and] pay one fee and see her three times and they are totally breast feeding moms now. But they were able to scrape it together with a partner that thought it was important to spend the money and drive up there. I just don't have a lot of people who are doing that.* (Participant #1)

Nearly all participants describe how one of their primary tools for supporting breastfeeding is a therapeutic use of self. They describe their caring contributions such as encouragement. According to one childbirth educator, “You know so much of it seems to me… is encouraging mother and supporting her in her choice and just continually reflecting back that she is doing a good job.” (Participant #2). And one staff nurse stated “… I end up spending hours working with the moms and …helping them with breastfeeding, trying to do a lot of encouraging.” (Participant #22). Many expressed empathy for their clients, as one nurse practitioner shared,

*… when I am working with mothers I don't seek to put pressure or make them feel bad for choices they make … I probably don't know fully what [it] is like, to feel like a mother who wants to breastfeed but who can't do it for some reason I think that must feel really, really challenging. Um, so I try to be sensitive to it.* (Participant #2)

And one staff nurse identified her client from her own experience, “But just that panic, you remember feeling that. So I guess I have empathy because I was kind of there before” (Participant #6).

The pattern of Operating within Constraints of the Particular Environment highlights the significance of the context within which HCWs and their clients live and work and how HCWs manage breastfeeding within existing constraints. Influences to breastfeeding support and services are in maternal, community, and healthcare spheres. Despite existing challenges, participants strive to make a difference with their clients.

### 5.4. Coexisting Messages

The pattern of* Coexisting Messages* (*n* = 466: 35%) refers to how mothers and HCWs are influenced by tacit or covert undercurrents that have an influence on breastfeeding and support efforts. Categories from all spheres of influence (i.e., maternal, family, community, and healthcare) emerged as part of this pattern.

A mother's internal attributes are described as an important influence on infant feeding decisions. This includes a mother's emotional state. Emotions such as feeling scared or insecure influence whether or not a mother seeks support for breastfeeding. One staff nurse shared her perspectives about maternal feelings of anxiety.

*They don't always know who they can call or sometimes maybe people don't want to feel like they don't know what they feel like maybe they should know? So just that I don't feel smart about this and I don't want to tell anyone. You know that scared that maybe I'm supposed to know this and I don't kind of feeling.* (Participant #6)

A community nurse reflected about maternal feelings of insecurity when she stated, “They're very insecure in their ability to do some of the things that you would think they might be more secure in, just their own body things.” (Participant #1). Several participants describe mothers as having a “mindset” of having preconceived plans for infant feeding which may or may not include breastfeeding. Some mothers are described as committed to breastfeeding regardless of any difficulties that they may face while others are less committed. According to one nurse practitioner,

*I think, too, that is another - it's not like a mindset and they're easy - to them it's the reverse. Like breastfeeding is optional, formula fed is the primary. It seems like that is more of the mindset until you can actually get in there and have a real effective conversation.* (Participant #14)

A preconceived plan that includes formula feeding can make efforts to encourage and support breastfeeding a challenge. Several participants said that if a mother really wanted to breastfeed and needed breastfeeding support, it is there and she could access it. Several expressed skepticism that their clients really want to breastfeed. One outreach worker stated, “I have some saying their [hospital] experience is not that great but then after talking with them for a while they'll kind of tell me I didn't really want to breastfeed” (Participant #18). A childbirth educator indicated that it is difficult to really know what many of the mothers plan to do. “I think most folks do plan on breast feeding that come through my classes or at least that is what they say” (Participant #2). Another childbirth educator felt that a maternal commitment to breastfeeding would overcome most hurdles?, “Mind you there are some females that are not capable of this for whatever reason, they might be a medical reason why they cannot but overall most women can if they have the mindset” (Participant #17). Several participants described mothers who breastfed successfully without any support, as one staff nurse expressed, “I mean you know I think a lot of women in the community are out there doing it and not getting formal services but they're doing a lot of reading on their own or kind of fending for themselves” (Participant #6).

Family circumstance and the family history of breastfeeding influence the family's understanding of infant feeding practices. Several participants described socioeconomic issues that the mothers and their families face as a barrier to accessing breastfeeding support. As one nutritional aide described the challenge,

*it's hard, really hard especially here where a lot of people live out off the grid. A lot of people here don't have a lot of money, they don't own cars; they don't have a cell phone that works all the time; they couldn't pay it. So how are they going to get to a phone because they don't have a car to get there and everything is far?* (Participant #16)

If breastfeeding is a common infant feeding practice in a mother's inner circle, a mother may be more inclined to breastfeed and seek support if needed. Participants described spouses or significant others and women's mothers and/or mothers-in-law as particularly influential. From one community worker,

*a lot of times you have a family, a mom, come in and have a baby that their family, their mom, was of the generation where they mostly bottle fed. So they're standing by that way side not really knowing how to encourage the mom.* (Participant #6)

Not knowing how to help may leave those closest to the mothers suggesting that they formula-feed their infants, “… it's just always easier to do this; it's always easier to give a bottle and have somebody else help you with your child.” (Participant #14). Many participants described the importance of spousal or significant other support for breastfeeding as essential. Some describe a negative perception among spouses and significant others about what type of woman breastfeeds her infant, “But, I know that there are guys that  don't want their wives to breastfeed because of this earth mother hippy dippy kind of connotation” (Participant #1). And one support group leader explained,

* I mean there are so many people who have doubting spouses, their partners that are - once you get that - I mean this is my helper, this is my mate and they're doubting that my body can do this, that I can feed our baby, you know. It's just intensely damaging to have that.* (Participant #7)

The culture and environment that mothers are immersed in guide their breastfeeding decisions. Participants described their experiences working with clients from different cultural backgrounds and how their understanding of culture informs their approach to support efforts. Modesty was mentioned by several participants as an important local value. From one program administrator,

*I think modesty is a huge one for local women. They're not - if they breast feed it's going to be in the car with a blanket over them because it's not something they're ever going to do publically at all.* (Participant #4)

And from another community worker,

*… having people who can visit in home and I think having people from different cultural backgrounds is key as well. I mean it is not just have the one lactation consultant in the area that looks like every, um, anyway, you got to have different people.* (Participant #2)

In addition to culture, participants described the influence of society and community expectations on the act of breastfeeding (i.e., in public) and breastfeeding decisions (i.e., not breastfeeding due to a negative perception about who breastfeeds). Many participants mentioned breastfeeding in public as an issue with mothers. One support group leader shared a conversation she had with another HCW in the community about support groups for breastfeeding mothers.

*She felt pretty strongly that the Hawaiian culture tends to be a lot more modest and to come into a setting where everybody is just openly breastfeeding and you might be in the middle of a conversation and forget to put your shirt down, she says it makes a lot of the people very, very uncomfortable. So why put yourself in a situation that is going to make you uncomfortable? So, yeah, there are definitely people who are turned off by the people that generally show up to a support group type of a setting.* (Participant #7)

Modesty or embarrassment about public breastfeeding was not limited to mothers but an issue for others in the community. From a medical assistant, “I mean they'll come in here and pop it out and all the other husbands are like freaking out. There are boobs everywhere. Well, it's a pediatrician's office” (Participant #12). And another WIC worker describes the situation in her office, “To make it - I know that sometimes our moms they breastfeed but they don't cover themselves and a lot of people are very uncomfortable when they don't cover themselves” (Participant #20).

Many participants describe formula feeding as the social norm or as one staff nurse put it “… the norm to bottle feed” (Participant #5) which hampers their support efforts. From one WIC staffer,

*formula fed is the norm, breastfed is like the bonus or something but it needs to be like it's a normal thing and I think it will be talked about more and maybe there will be a lot more women that will want to persevere because it's a normal thing to do.* (Participant #18)

HCWs described how they bring their experiences, culture, and background, whether local or from the mainland, to their practice. These are tacit or covert influences and stem from their frame of reference, work setting, or beliefs about breastfeeding. Several of the participants had work experiences on the mainland and readily compared and contrasted them to what they feel is happening in Hilo. One staff nurse observed

*coming from an area that had a lot of baby friendly hospitals, I mean, when you went to the movie theater it would advertise breast feeding before the movie. There would be posters up at the mall… I don't think that is what the community sees here.* (Participant #5)

Another participant shared her experience.

*But where I trained in [on the mainland], we had several lactation consultants on staff and if a mom wanted to breast feed I never saw them fail. I mean it just happens. They're just really good at what they do usually.* (Participant #8)

Other participants did not have experiences beyond Hawai‘i and had a different perspective. An administrator reflected about changes that are gradually taking place in Hilo.

*I guess only that things have been improving. I've been here for quite a while, over 20 years. It's much better than it was 20 years ago as far as the kind of training that we've had and the support group breastfeeding.* (Participant #19)

Many of the participants with local experience rely more on their own anecdotal experience rather than that of previous work settings. A staff nurse described how she approaches assisting mothers.

*Then I just like, hey, just relax, I'll do it. I position them. I love that. I just love it. They're like, oh, my god, thank you…I know it because I know how I wanted to only breastfeed, you know.* (Participant #23)

And from another outreach worker, “Personally for me I got the support from my doctor and I have the support at work and I want to breastfeed. I wanted to educate myself on other things I didn't know about or any problems” (Participant #18).

The majority of participants voiced a commitment to supporting breastfeeding but their descriptions of their knowledge level varied. Many felt the need for more education. One provider stated, “I feel basically clueless about breast feeding… Basically, I feel kind of sad when I can't really help families” (Participant #8). An outreach worker had a similar perspective, “I answer them as best as I can. If I feel that there is something I can't answer I will find the answer out for them but also refer them to somebody who can help them” (Participant #18). And one of the CLCs from WIC described his frustration.

*Sometimes it can be frustrating because I do know the limitations…that we really have nobody in this clinic who is really comfortable looking at latches and that is like the number one problem.* (Participant #21)

The pattern of* Coexisting Messages* was defined as the tacit or covert messages that influence mothers and HCWS. These undercurrents include what mothers and HCWs bring to breastfeeding decisions and practices. All spheres of influence emerged as part of this pattern.

### 5.5. Process Interrupted

This pattern of* Process Interrupted* (*n* = 289:  22%) refers to a breakdown in the process of supporting breastfeeding. Despite best efforts of HCWs, messages  do not get through or resources are missing. Maternal and healthcare sphere are represented in this pattern.

Accurate and consistent information about breastfeeding does not always reach mothers due to a variety of reasons including maternal factors (i.e., situational), the messenger, or the message (i.e., lack of or altered). Several participants described how inconsistent messages from HCWs can be confusing for mothers. From one community lactation counselor,

*because you hear different things from everybody and maybe that's normal to some extent. You're going to have different people have different ways of saying things but to have what, often I hear from mothers, well, three different people told me three different things and I did not know what to do. Um, Oh, “People tell me to feed my baby every three hours and you know then somebody else says every 5 and you know there's just different things that get said.”* (Participant #2)

The fatigue and sensitivity of mothers following childbirth was described as a vulnerable time when mixed messages can be upsetting to mothers. According to one staff nurse,

*so you come into your patient, they were told by the nurse last night that, oh, having a bottle and pacifier is totally fine and that somebody else told them it was going to be a problem and now you've received them and they're crying because they don't know what to do.* (Participant #5)

The educational level of mothers was identified by several participants as an indicator of whether or not a mother chooses to breastfeed or feels confident enough to seek out help about breastfeeding. According to one nurse practitioner, “There definitely seems to be an educational aspect to that, too. The more educated the more likely they are to breastfeed” (Participant #13). Prenatal education about breastfeeding was also identified by many participants as important. Many described how they support breastfeeding prenatally, as noted by one community nurse.

*We hand them [breastfeeding information] out appropriately time to their pregnancy and their questions. I think that it's really helpful for them to have it in hand when they're at home thinking about what they want to do when this baby and feeding it and stuff like that.* (Participant #1)

Many participants talked about how difficult it was to initiate their first discussions about breastfeeding in the postpartum period. The prenatal period was mentioned as a good time to start discussing breastfeeding. According to one participant,

*education prior because that decision has to be made before she gives birth to a certain extent. Must have some commitment going into it because it's such a messy time that I think if you, it's something you haven't even thought about you're probably not going to put a lot of energy into it right then.* (Participant #4)

Another aspect to breastfeeding education identified by participants was the source of information. In some cases, HCWs felt that the message carries more weight depending on the messenger. One medical assistant related this from her personal experience.

*…I was up there with my daughter and she didn't want to breastfeed and they just said okay, sure. I wish they would have just persuaded her and educated her a little bit more about how healthy it is. I was right there but she ended up not breastfeeding anyway. She's not going to listen to me but maybe if somebody else would have like told her.* (Participant #11)

Another participant had a similar perspective.

*sometimes they'll take it more into consideration if they hear it from somebody else than their mom. They think, oh, their mom is telling them what to do again, you know, rebellious. So the mom will come up to us and say I've been showing her how to breastfeed but she's not listening to me can you tell her this and that.* (Participant #12)

Other participants described how getting information from an authority figure may not be the best way to assist with breastfeeding. A certifier for the WIC Program shared her perspective about this.

*…this is not everyone but I don't feel like a lot of the ones that are the younger ones like they don't really want to listen to someone in authority telling you, you do it this way. I think they feel more comfortable hearing like, oh, my sister does it this way, oh, my best friend likes to do it.* (Participant #17)

### 5.6. Healthcare Sphere

HCWs identify many missing components in the system that could help support breastfeeding. Some participants clearly identified the lack of resources as being a major problem. One office staff member observed, “… outside of this office there is not very many supports for breastfeeding” (Participant #1). Another staff member shared similar feelings about the limited resources, “What else is there? Other than WIC off the top of my head I cannot really think” (Participant #14).

One of the most frequent observations shared was the need for lactation consultants in both community and hospital settings. One community nurse stated

*…why the hospital here doesn't have lactation consultants, it could be any one of those reasons: They  don't think it's important, they  don't think they can afford it, they're not really exploring, the doctors  don't want to open up and let anybody else come into do this work. They have to answer for that, I really  don't know.* (Participant #1)

A hospital staff member reflected about the need for expert consultation to be available in the hospital setting.

*I think that we should have a lactation consultant associated with the hospital, not just a name on a card that none of us has ever meant in the community that I'll give out this number from our rolodex. I  don't even know if this person is still in practice. I  don't know who updates this information. So somebody that is actually affiliated with the hospital that we know is up-to-date on their information that can follow-up with these patients.* (Participant #5)

Many expressed concern regarding the lack of follow-up for mothers. Participants working in inpatient settings described sending parents home, as one shared.

*So we're sending home these babies that are breastfeeding with no follow-up. Usually they can't leave the hospital unless they have some sort of follow up plan within two to three days but a lot of times we want to do the weight check within one or two days. If you're discharging them on a Friday there is no follow-up on the weekend.* (Participant #5)

Participants also describe situations where HCWs could have helped and encourage breastfeeding but did not or were unable to support mothers. Many of these comments stemmed from care in the hospital. Frequent statements focused on system barriers such as staff being too busy to help mothers or hospital practices that are not conducive to breastfeeding. One pediatrician observed, “Lactation consulting takes time. It's time intense. If the ward is full of new moms and new babies that can kind of get away from it” (Participant #8).

Another participant reflected about the lack of breastfeeding support by nursing staff.

*I think they…sense if the nurses are busy and they're bothered, you know, if they're helping them. I think they kind of pick up on that and they're afraid to ask for help. And then they come in and when did the baby last feed and then they get scold them because the baby has not eaten.* (Participant #10)

Hospital staff were generally described as knowledgeable about breastfeeding but the availability of anyone to help on regular basis was lacking. One community nutritionist described the limited access to breastfeeding assistance.

*They're delivering they ask, we tell them ask for the - there is a lactation consultant, I think, in the building. We tell them to ask, they ask and maybe one or two have said oh, yeah, someone has helped me but the rest are, oh, no, nobody was in, I think they weren't working that day I gave birth.* (Participant #15)

The pattern of* Process Interrupted *encompasses multiple aspects related to breastfeeding supports and services including the lack of consistent information or resources for mothers.

## 6. Discussion

Breastfeeding supports and services in Hilo, Hawai‘i, were explored using ethnographic methods, to describe HCWs' understanding of community breastfeeding supports and services that exist within Hilo, Hawai‘i. The three patterns that emerged from the analysis,* Operating within Constraints of the Particular Environment, Coexisting Messages,* and* Process interrupted, *have aspects consistent with existing literature as well as aspects that are unique to the specific context in which this research was conducted. The patterns, although discussed separately, are parts of a whole.

### 6.1. Operating within Constraints of the Particular Environment

One of the recurring themes that emerged was of the healthcare challenges of this rural island setting. The following section includes a discussion of the socioeconomic difficulties, geographic challenges, and cultural issues which influence communication and the delivery of care in the community.

### 6.2. Geography

HCWs describe how they are constrained by the existing healthcare structure and try to manage with limited resources. These observations are similar to what other HCWs have described working in rural communities [27]. Rural settings offer challenges in the provision of healthcare and historically have had poorer quality healthcare than more urban areas [28]. Many participants described cost as the reason for the lack of breastfeeding support resources. Without competition there is nothing compelling facilities or practices to allocate resources to a lactation program except consumer demand and federal policy and mandates. The recent Call to Action from the Surgeon General has specific recommendations for facilities including improving access to IBCLCs and the provision of breastfeeding related education for those working with mothers and infants [6]. These recommendations may serve as an impetus for change. However, the economic recession is a continued challenge to area healthcare facilities and has in some cases led to budget cuts and staffing furloughs and reductions. Despite recent reports that the population of Hawai‘i County grew the fastest of any other county in the state in 2010 (an increase of 24.5%) [29], jobs in the healthcare and social service sectors of the county dropped by 200 in 2010 [30].

### 6.3. Socioeconomics

The island population served by this group of HCWs is characterized by lower income and higher unemployment than other more urban areas of the state. In a recent release of an annual health rankings of counties Hawai‘i County ranked as the unhealthiest county in the state with the highest percentage of children living in poverty and the highest rate of teen pregnancy among other indicators [31]. It is known that early childhood experiences such as slow growth become embedded in biology during development and may affect health throughout the lifetime [32]. It is not known if and how breastfeeding promotion and support could improve health outcomes for the vulnerable groups in this island community but it seems likely if support results in longer breastfeeding duration.

### 6.4. Communication Style

Two different approaches to communicating about healthcare issues on the island emerged from participants as they shared their observations. First, some of the HCWs interviewed expressed acceptance about the current state of healthcare on the island and hesitated to criticize other institutions or practitioners. They expressed recognition that some things are lacking but said they do their job as best as they can with what they have, without challenging the status quo. With the second communication approach, HCWs more readily verbalized what is lacking and described how they tried to address issues in their workplace or community. The difference in the way that participants communicated their views suggests that there may have been some cultural influences reflective of the differences between different ethnic groups.

In general, people from individualistic cultures place greater emphasis on the informational aspects of communication while people from collectivistic cultures place greater emphasis on the relational function [33]. Collectivism is a common cultural pattern in Asia, Africa, Latin America, the Middle East, and the Pacific. It recognizes the needs of the group superseding the needs of the individual. Individualism by contrast centers more on independence and self-reliance and is a common cultural pattern found in European and North American countries [34]. Participants with an individualistic perspective may see themselves as more independent rather than interdependent; they value verbal clarity and individual opinion, which may explain why they express more difficulty accepting the existing healthcare situation.

The way cultural groups perceive the world could play a role in communication. According to an author and long-term resident in the community, an old and frequently heard local Japanese saying,* the nail that sticks out gets hammered down*, epitomizes why some participants may have been less likely to criticize the existing healthcare system (P. Iwasaki, personal communication, February 16, 2011). Speaking out may not be a value. For those who had practiced on the mainland prior to working in Hilo, many expressed frustration or exasperation with what they see as lacking in the current healthcare situation. This difference in approaches may be an influence in the difficulty described by some participants when pushing for reforms in the existing system and leadership gaps.

### 6.5. Coexisting Messages

The influences on infant feeding decisions are numerous and have been explored in the literature. Supportive or unsupportive messages can be overt or covert and exist more as an undercurrent. These tacit or implicit messages may be more influential in the infant feeding decisions mothers make. In this exploration, participants described instances where tacit knowledge influenced their practice and mothers breastfeeding decisions.

Participants bring to their work a frame of reference that includes practice experiences from previous work settings and their own anecdotal experiences with infant feeding [35]. Lack of knowledge about how to manage lactation problems or not knowing where to send mothers for help was an issue. The lack of resources for breastfeeding mothers also sends a mixed message to mothers. If no one in an office or clinic has the skills to help a mother breastfeed or does not know where to refer a mother, what kind of message is sent [36]?

Tacit or covert messages also influence mothers in their infant feeding decisions. Consistent with what others have found, mothers were described as being influenced by those closest to them [37, 38]. Mothers, mothers-in-law, and significant others were part of an influential circle consistent echoing other research findings [15, 16, 39]. Many participants describe issues with young mothers reflecting that breastfeeding may be incompatible with a teen's lifestyle. Peer influence also contributes to young mothers' feeding decisions [40]. A culture of formula feeding was described as the norm among some young mothers. Several participants described a negative societal perception of breastfeeding in the community: in particular, breastfeeding was a “hippy dippy” thing to do. Social or cultural norms have been identified as an influence breastfeeding decisions [15, 41] and in the US, bottle feeding is often seen as the “normal” way to feed infants ([6], p. 11).

Many described breastfeeding in public as offensive or uncomfortable. One of the participants described how a group seeking to promote breastfeeding in the community staged a breastfeeding protest at the local library. A photo appeared in the newspaper which showed the group openly breastfeeding in front of the library. The participant posted the newspaper clipping above her desk at her work site. One of the mothers, who came in to the office, pointed to the photo and said that this was why her husband would not let her breastfeed. A stereotype of who is breastfeeding and the way that some women openly breastfeed in public may contribute to a negative view of breastfeeding by some in the community. Concerns of modesty and public breastfeeding were mentioned by participants and echo other research findings related to breastfeeding in Hawai‘i [37, 38, 42].

Mothers were described by some participants as having a preconceived plan for infant feeding: a mindset to either breastfeed or formula-feed. Since their mind was already made up, it was felt that no intervention to support breastfeeding would change the mother's mind. Several said that if a mother really wanted to breastfeed, she would. If she wanted to find help, she could. This places the onus of responsibility on mothers who may not know where to seek help. A lack of knowledge about the benefits of breastfeeding or how to manage and learn breastfeeding skills has been identified as influence on breastfeeding decisions [43, 44]. Future research could include a deeper exploration of the support interventions utilized by HCWs in the community and learn more about what they find effective. In addition, including the mothers' voices may shed more lights on what is motivating their breastfeeding decisions.

### 6.6. Process Interrupted

Ideally, breastfeeding support is seamless beginning in the prenatal period, extending through the hospital delivery birth experience with follow-up provided for the new mother once she is home [6]. Some of the participant HCWs (i.e., WIC staff) work with mothers, prenatally and through the postpartum periods, but most participants saw mothers during either the intrapartum or the postpartum period. Many of the participants who work in the hospital setting described mothers as having already made their minds up regarding their infant feeding plans when they come to deliver their infants. They expressed the need for more prenatal education. Participants who worked with mothers postpartum described issues with the hospital and lack of lactation staff as having a negative impact on mothers' breastfeeding efforts. Ultimately both groups are correct, there is no crucial time period, and all periods are important: prenatal, intrapartum, and postpartum [6, 45, 46]. Each HCW may only be aware of what is occurring in their respective job setting which may lead them to identify the issues or problems in other settings. This highlights the need for more continuity of care in the community and better communication between the HCWs working in different settings (i.e., hospital and clinics).

If mothers receive breastfeeding information during the prenatal period, they are more likely choose to breastfeed and be better prepared to make the transition to nursing [47]. Another crucial time when breastfeeding support and services could make a difference is during hospitalization for delivery. The importance of early attachment at the breast has been well documented and current recommendations are for mothers to breastfeed in the first half hour following delivery [48]. Participants described hospital routines and practices that keep infants separated from mothers following delivery preventing early breastfeeding experiences which interfere with the establishment of breastfeeding. Breastfeeding support was described as inconsistent. Inadequate staffing, early discharge, and inconsistent breastfeeding information from staff were identified as issues at the hospital. The pervasiveness of formula use in the hospital was another issue raised. In one study in Hawai‘i, Pager et al. [49] found that early use of formula in the hospital was associated with an early cessation of breastfeeding. The majority of participants said that having an IBCLC(s) at the hospital would help. Castrucci et al. [50] found that infants are more likely to be breastfeeding on hospital discharge if the facility employs an IBCLC. The hospital IBCLC role is to teach classes, provide training to staff, make rounds on breastfeeding mothers, and provide follow-up. The current recommendation for staffing ratios for IBCLCs in hospitals is one IBCLC per 1000 live births [51]. The hospital in Hilo averages 1200 births per year and does not have any IBCLCs on staff.

The days and weeks following delivery are a critical time period for a breastfeeding mother. Data from WIC on the reasons for weaning in Hilo showed the majority of mothers who weaned did so in the first four weeks following delivery. Issues given such as low milk supply or pain could be addressed and managed with skilled follow-up. Bonuck et al. [52] found that women who receive postpartum visits and telephone follow-up from a lactation consultant are more likely to breastfeed through 20 weeks. However, postdischarge support for breastfeeding is not only a problem in Hilo but also a statewide problem. This is highlighted in the mPINC survey of breastfeeding related maternity practices at hospitals in the US; Hawai‘i ranked lowest score in the nation, receiving a score of 14 out of a possible 100 for breastfeeding support after discharge [7]. As previously mentioned, it is not known how Hilo Medical Center ranked within the state. However, many of the participants, in hospital and community settings, identified maternity practices incongruent with support for breastfeeding (i.e., formula supplementation).

The breastfeeding support process extending from the prenatal to postpartum period has numerous gaps, resulting in lost opportunities to support breastfeeding. Communication and collegiality between HCWs could help bridge the gap between the prenatal period, hospital, and home. Some participants said they did not always know when their clients delivered and that many times mothers quit breastfeeding before they were seen. This highlights the importance of support during the early weeks of breastfeeding which may prolong breastfeeding [53].

## 7. Conclusion 

A predominate thread flowing through all parts of this research is communication. Communication between different cultural groups and between different healthcare settings in the community influence care delivery. Consistent messages from HCWs in all settings supporting breastfeeding from the prenatal period through postpartum are needed. Participants were in agreement on the importance of family members in infant feeding decisions.

The findings of this study reinforced Hilo as a rural semi-isolated region with limited resources to support breastfeeding. Existing options for support are primarily informational due to limitations in qualified lactation personnel. The geographic and socioeconomic characteristics of the area make it difficult to offer or expand services. Educational opportunities and experiences to improve breastfeeding support skills for community HCWs are limited. This creates a challenge of how to maximize existing resources in a sustainable and culturally sensitive way. This community of HCWs is engaged and interested in supporting breastfeeding. The next steps could include working with the community to identify solutions that make sense for Hilo.

### 7.1. Future Directions in Research

More research is needed to understand how to address this community's breastfeeding support and service needs. The second phase of this research is to include the voices of breastfeeding mothers in the community thereby providing triangulation about the findings. This would add to the understanding of the breastfeeding support and service needs specific to mothers living in Hilo. Additionally, a quantitative exploration of breastfeeding rates in Hilo would shed light on the accuracy of existing government data set reports of breastfeeding initiation and duration. This could include a hospital chart review to find out exactly how much breastfeeding is occurring in the hospital and how much supplementation is involved. Finally, deeper exploration of breastfeeding in other areas of the island could help explain the differences in breastfeeding rates.

### 7.2. Limitations

The sample does not mirror the population demographics but may reflect the demographics of HCWs: largest group, Caucasians. This group of HCWs was homogeneous in the sense that they all work with the same population, but they varied in role, educational background, and experience. Those that chose to participate may have been pro breastfeeding and other HCWs who were less supportive or had a different perspective on infant feeding may have opted not to participate. Researcher positionality is an important limitation. Participants knew the focus of the research was breastfeeding support and that the researcher had a background in lactation support, so they may have stated what they thought the researcher wanted to hear or would come across as supportive of breastfeeding. Although the researcher has lived in Hawaii more than 10 years, she is Caucasian and not a local long-term resident. Additionally there is the power differential that some participant responses may have been affected by the difference in education level or job position between the researcher and participant. Finally, the results may not be transferable to other groups; ethnographic transferability is dependent on a specific time and context.

## Figures and Tables

**Table 1:** Patterns found in HCWs understandings of community breastfeeding supports and services.

Patterns	Categories
Operating within Constraints of the Particular Environment (*n* = 584: 44%)^a^	(i) Situated context
	(ii) Geography matters
	(iii) Structure creates hurdles
	(iv) It is how we do
	(v) Caring contributions
	(vi) Making the most of what exists

Coexisting Messages (*n* = 466: 35%)	(i) Maternal inner space
	(ii) An influential circle
	(iii) Situated family life
	(iv) It is customary
	(v) Tacit understandings affect practice

Process Interrupted (*n* = 289: 22%)	(i) Message transmission failure
	(ii) Lost opportunities
	(iii) Missing linkages

^a^The numbers given are the numbers of quotes and the percent is the percentage of the entire number of quotes.
